# High-throughput Microwell-seq 2.0 profiles massively multiplexed chemical perturbation

**DOI:** 10.1038/s41421-021-00333-7

**Published:** 2021-11-09

**Authors:** Haide Chen, Yuan Liao, Guodong Zhang, Zhongyi Sun, Lei Yang, Xing Fang, Huiyu Sun, Lifeng Ma, Yuting Fu, Jingyu Li, Qile Guo, Xiaoping Han, Guoji Guo

**Affiliations:** 1 Center for Stem Cell and Regenerative Medicine, and Bone Marrow Transplantation Center of the First Affiliated Hospital, Zhejiang University School of Medicine, Hangzhou, Zhejiang China; 2 Liangzhu Laboratory, Zhejiang University Medical Center, 1369 West Wenyi Road, Hangzhou, Zhejiang China; 3 School of Basic Medical Sciences, Zhejiang University School of Medicine, Hangzhou, Zhejiang China; 4 Zhejiang Provincial Key Lab for Tissue Engineering and Regenerative Medicine, Dr. Li Dak Sum & Yip Yio Chin Center for Stem Cell and Regenerative Medicine, Hangzhou, Zhejiang China; 5 Institute of Hematology, Zhejiang University, Hangzhou, Zhejiang China; 6 ZJU-UOE Institute, Zhejiang University, School of Medicine, Haining, Zhejiang China

**Keywords:** Biological techniques, Cell biology

Dear Editor,

Cell-based high-throughput screening (HTS) is an important strategy for discovering a new medicine^1^. Assays suitable for HTS should be sensitive, robust, and economical. However, the readout of conventional HTS assays is restricted to gross phenotypes, including bulk transcriptional profiles, fluorescence signals, morphology, and viability, which cannot reveal subtle and heterogeneous changes in individual cells. In recent years, high-throughput single-cell sequencing technology has shown promise in overcoming these limitations in cell-based HTS. For HTS, single-cell RNA sequencing (scRNA-seq) has been combined with several cell-labeling strategies, including cellular hashing (e.g., sci-Plex^2^) and CRISPR/Cas9 (e.g., Perturb-Seq^3^). In addition, in-cell reverse transcription (RT) reactions can label cells using barcoded primers and significantly increase the throughput of scRNA-seq^4–6^. Our previous works of mouse cell atlas^7^ and human cell landscape^8^ showed that Microwell-seq 1.0 is a sensitive, robust, and cost-effective scRNA-seq technology with advantages of low batch effects and high cell-type compatibility. Combining in-cell RT and Microwell-seq 1.0, we established Microwell-seq 2.0 for cost-effective and high-throughput HTS with single-cell transcriptional profiling (Fig. 1a; Supplementary Fig. S1).

**Fig. 1:**
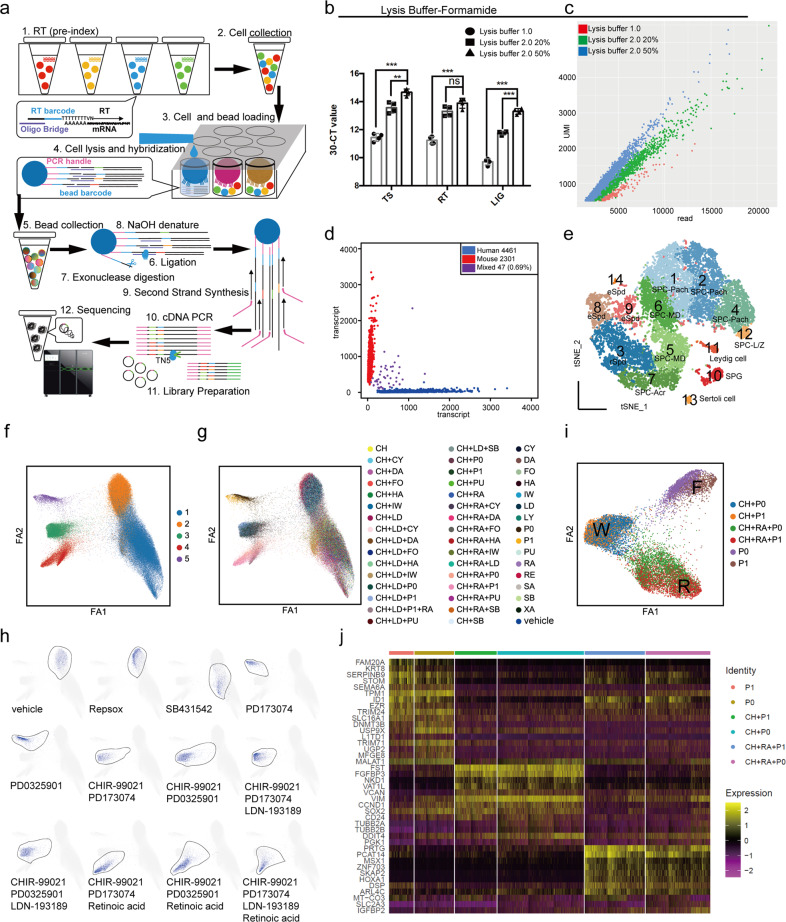
High-throughput Microwell-seq 2.0 enables multiplexed chemical perturbations. **a** Schematic diagram of Microwell-seq 2.0. **b**, **c** qPCR and NGS analysis using three lysis buffers, respectively: Microwell-seq 1.0 lysis buffer, 2.0 lysis buffer (with 20% and 50% Formamide). Data are means ± SD, *n* = 4; *P* values were calculated by Student’s *t*-test; ns, no significance; **P < 0.01, ***P < 0.001 (**b**). Scatter plot of NGS data shows the transcript number versus the read number of each individual cell (**c**). A summary of NGS was listed in Supplementary Table S6. **d** Scatter plot of human–mouse mix test using Microwell-seq 2.0. Only 0.69% (purple dots) are human–mouse mixed cells. **e** t-SNE map of adult mouse testis analyzed by Microwell-seq 2.0. Cells are colored by cell-type cluster. SPG, spermatogonia; SPC-L/Z, spermatocyte-leptotene/zygotene; SPC-Pach, spermatocyte-pachytene; SPC-MD, spermatocyte-meiotic division; SPC-Acr, spermatocyte-acrosomal phase; rSpd, round spermatid; eSpd, elongating spermatid. **f**−**h** PAGA graphs show the potential cell transitions in chemical perturbation. Five cell-type clusters were labeled in the graph generated by ForceAtlas2 (FA2) (**f**). Forty-eight small-molecule combinations were labeled in the graph generated by FA2 (**g**). PAGA plots show cell distribution after treatment with different small-molecule combinations (**h**). **i** PAGA graph shows the potential cell transitions in perturbation of CHIR-99021 (CH), PD173074 (P1), PD0325901 (P0), and Retinoic acid (RA). **j** A gene expression heatmap shows top differentially expressed genes for small-molecule combinations in **i**. Yellow corresponds to high-expression levels; purple and black correspond to low-expression levels.

We carried out a series of optimizations to considerably improve the sensitivity of Microwell-seq 2.0. We established a TaqMan qPCR-based optimization system to speed up the process and dramatically reduce the cost (Supplementary Fig. S2). The CT value was used for preliminary evaluation of different reaction conditions, and next-generation sequencing (NGS) was used for verification. In the workflow of Microwell-seq 2.0, cells were first fixed and barcoded (round 1) in RT reactions using well-specific RT primers, corresponding to the given perturbations. We tested two RT temperatures (42 and 55 °C), of which 42 °C-RT had a higher cell recovery rate (Supplementary Fig. S3a, b). In 55 °C-RT, the cells were sticky and hard to collect and load. For in-cell RT, reverse transcriptase needs to be resistant to inhibitors that may carry over from fixation and complex intracellular environments. Maxima RTase showed the highest sensitivity, consistent with previous work^5^ (Supplementary Fig. S3c). One hour of incubation at 42 °C was necessary for the RT reaction. Additional 42 °C incubation and plate-rotation did not significantly increase the RT efficiency (Supplementary Fig. S3d, e). Furthermore, we found that the best sensitivity was obtained using 25 T poly-T primers (with and without -VN) (Supplementary Figs. S3f, g, S4c, d). We found that 1 M betaine did not improve the RT efficiency (Supplementary Fig. S3h). We also replaced KCl in Maxima RT buffer with NaCl, which improved RT sensitivity as previously reported^9^ (Supplementary Fig. S3i). After RT pre-indexing, all cells were pooled and loaded into the agarose plates. In Microwell-seq 1.0, an agarose plate with 10^5^ microwells was used, which can trap only 10,000 individual cells per experiment. To load multiple cells, we increased the aperture and depth of the microwells (Supplementary Figs. S1e, S3j). Moreover, we adopted a honeycomb-like arrangement to reduce the space gap so that each plate can accommodate more microwells. An agarose plate of Microwell-seq 2.0 with 70,000 wells can contain up to 700,000 individual cells, which can meet the demand of high-throughput screening. The optimization of the microwell plate also improved the adaptability for various cell types with different sizes. After cell loading with centrifugation, most of the wells were filled, and multiple cells occupied the same well (Supplementary Fig. S1f). Then, barcoded magnetic beads were loaded and trapped into most of the wells. After cell and bead loading, lysis buffer was used for cell lysis and hybridization. Formamide-based lysis buffer (2.0 lysis buffer) was more suitable for hybridization of DNA–DNA than 1.0 lysis buffer (Fig. 1b, c; Supplementary Figs. S3k, S4a, b). Hybridization with 50% formamide and 5× SSC improved the sensitivity. Neither T4 buffer nor PEG resulted in better hybridization (Supplementary Fig. S3l, m). Barcoded oligonucleotides on the beads captured and labeled cDNA (round 2) by ligation. Then, we tested three ligation systems: Ampligase, T4 ligase, and *E. coli* ligase (Supplementary Fig. S3n). We chose T4 Ligase, which can ligate hybridization substrates with 1–2 nt gaps. After ligation, it was necessary to digest the bead oligonucleotides that did not capture cDNA (Supplementary Figs. S3o, S4e, f). To add the PCR handle for cDNA amplification, we performed second-strand synthesis^10^. Maxima RT buffer performed better than Klenow Exo- buffer in second-strand synthesis, consistent with previous work^10^ (Supplementary Fig. S3p). To prevent multiple displacement amplification (MDA), excess dN-TSO primer was removed before polymerization of Klenow Exo- (Supplementary Figs. S3q, S4g, h). After second-strand synthesis, barcoded cDNA was enriched by PCR and fragmented by customized Tn5 transposase with two identical insertion sequences. To reduce sequencing costs, we sequenced the linear 3ʹ ends of the transcripts using the MGI DNBSEQ-T7 platform. After sequencing, the transcriptome of individual cells was assembled by combining reads containing the same two-barcode combination. Our work observably improved the sensitivity, robustness, and economic efficiency of Microwell-seq 2.0.

To assess the fidelity of Microwell-seq 2.0, we performed a species-mixing experiment with cultured human (293T) and mouse (3T3) cells. After second-strand synthesis, 1/10 beads were used for cDNA amplification and library sequencing. With shallow sequencing, we obtained 6809 cells (mean UMI 739, mean gene 592, mean read 1117) with no more than 0.7% cell doubles (Fig. 1d). Moreover, we assessed the platform on tissue cells with more heterogeneous cell types. One mouse testis was processed using Microwell-seq 2.0 (Fig. 1e; Supplementary Table S1 and Fig. S4i). We obtained 12,363 cells (mean UMI 839, mean gene 680) and identified 14 cell types, including spermatogonia (SPG), spermatocyte (leptotene/zygotene, pachytene, meiotic division, and acrosomal phase), spermatid (round spermatid and elongating spermatid), leydig cell, and sertoli cell. The germ cell clusters formed a typical wave-like continuum. Notably, Microwell-seq 2.0 showed advantages in sensitivity and robustness over other scRNA-seq approaches (Supplementary Fig. S4j, k and Table S2).

By harnessing the power of Microwell-seq 2.0, we analyzed massively multiplexed chemical perturbation of human embryonic stem cells (hESCs) at single-cell resolution. We selected 16 small molecules widely used to target the key pathways in stem cell biology (Supplementary Table S3). We exposed H9 cells (hESCs) to each of 48 combinations for 48 h in duplicate (Supplementary Table S4). Cells from each well were fixed separately and subjected to in-cell RT for cell labeling followed by single-cell transcriptional profiling using Microwell-seq 2.0. After sequencing and filtering, we obtained 108,782 single cells (mean UMI 536, mean gene 454, mean read 1169). We used uniform manifold approximation and projection (UMAP) to visualize these data and defined five clusters with specific markers (Supplementary Fig. S5 and Table S5). Small-molecule combinations were specifically distributed in five clusters (Supplementary Fig. S6). Next, we used partition-based graph abstraction (PAGA) to show cell transitions in chemical perturbation (Fig. 1f–h; Supplementary Figs. S7, S8). Both Repsox and SB431542 are ALK inhibitors (Repsox: ALK5, ALK4, ALK7; SB431542: ALK5, TGFβR1). Microwell-seq 2.0 sensitively identified their different perturbation effects (Fig. 1h). PD173074, PD0325901, CHIR-99021, and retinoic acid played important roles in the spread of branches 3, 4, and 5 (Fig. 1h, i; Supplementary Fig. S7). The FGFR inhibitors (PD173074 and PD0325901) induced the expression of *FGFR3* in cluster F. With the perturbation of CHIR-99021 (Wnt/β-catenin activator), cluster F switched to cluster W with the expression of *VIM* (a general marker of mesenchymal fate), *FST* (a marker of myogenic differentiation), *FGFBP3*, and *CCND1* (canonical Wnt/β-catenin transcriptional target) (Fig. 1j). With the perturbation of retinoic acid, cluster W switched to cluster R with the expression of *SKAP2* (retinoic acid-induced protein 70) and *PRTG* (a marker of neuroectodermal development). Some small molecules (such as CHIR-99021) alone can significantly affect gene expression. However, some small molecules (such as retinoic acid) need to be combined with others to produce obvious perturbations. Multiplexed Microwell-seq 2.0 enables a detailed molecular dissection of chemical perturbations during hESC differentiation with complex small-molecule combinations.

The pre-index strategy with Microwell-seq is not limited to scRNA-seq. We show that the same method can be used to enhance the throughput of single-cell ATAC-seq for HTS. Here, we also established Microwell-2.0-ATAC-seq (Supplementary Fig. S9) with a potential for multimodal HTS.

In summary, these results illustrated the high sensitivity and robustness of Microwell-seq 2.0 in cell-based screening. Our method may pave the way for a more cost-effective multi-dimensional and high-throughput drug screening assay.

## Supplementary information

Supplementary Information

Supplementary Table S1

Supplementary Table S2

Supplementary Table S3

Supplementary Table S5

Supplementary Table S6

Supplementary Table S7

Supplementary Table S8

Supplementary Table S9

## Data Availability

All raw and processed datasets are available from the NCBI GEO database (GSE175413).

## References

[1] Blay, V., Tolani, B., Ho, S. P. & Arkin, M. R. High-throughput screening: today’s biochemical and cell-based approaches. *Drug Discov. Today*10.1016/j.drudis.2020.07.024 (2020). 10.1016/j.drudis.2020.07.024 32801051

[2] Srivatsan SR (2019). Massively multiplex chemical transcriptomics at single cell resolution. Science.

[3] Dixit A (2016). Perturb-Seq: dissecting molecular circuits with scalable single-cell RNA profiling of pooled genetic screens. Cell.

[4] Cao J (2019). The single-cell transcriptional landscape of mammalian organogenesis. Nature.

[5] Rosenberg AB (2018). Single-cell profiling of the developing mouse brain and spinal cord with split-pool barcoding. Science.

[6] Datlinger P (2021). Ultra-high-throughput single-cell RNA sequencing and perturbation screening with combinatorial fluidic indexing. Nat. Methods.

[7] Han X (2018). Mapping the mouse cell atlas by Microwell-Seq. Cell.

[8] Han X (2020). Construction of a human cell landscape at single-cell level. Nature.

[9] Hagemann-Jensen M (2020). Single-cell RNA counting at allele and isoform resolution using Smart-seq3. Nat. Biotechnol..

[10] Hughes TK (2020). Second-strand synthesis-based massively parallel scRNA-Seq reveals cellular states and molecular features of human inflammatory skin pathologies. Immunity.

