# Supplementary material for: High-throughput Microwell-seq 2.0 profiles massively multiplexed chemical perturbation
Source: Cell Discov. 2021 Nov 9;7:107. doi: 10.1038/s41421-021-00333-7 (PMC8575926; doi:10.1038/s41421-021-00333-7)
Supplement: Supplementary file 1 — Supplementary Information [file 41421_2021_333_MOESM1_ESM.pdf]

SUPPLEMENTARY INFORMATION for

High-throughput Microwell-seq 2.0 profiles massively multiplexed chemical perturbation

Haide Chen[1, 2, 3, 4, 5], Yuan Liao[1, 3, 4, 5], Guodong Zhang[1, 3], Zhongyi Sun[1, 3], Lei Yang[1, 3], Xing Fang[1, 4], Huiyu Sun[1, 3], Lifeng Ma[1, 3], Yuting Fu[1, 3], Jingyu Li[1, 3], Qile Guo[6], Xiaoping Han[1, 3, 4, 5], Guoji Guo[1, 2, 3, 4, 5 *]

This file includes:

Materials and Methods

Supplementary Figures S1 to S9, and legends

Supplementary Table S4 and S10

References

Materials and methods

Preparation of cell suspensions

293T and 3T3 were cultured in Dulbecco's Modified Eagle Medium (DMEM, Thermo) supplemented with 10% Fetal Bovine Serum (FBS, Thermo) and 1% penicillin-streptomycin (Thermo). Cells were cultured in the 6-well culture plates (Corning) in an incubator with humidified air and 5% CO_2 at 37°C. Cells were harvested by trypsinization and resuspended in cold Dulbecco's Phosphate-Buffered Saline (DPBS, Corning).

Wild-type C57BL/6J male mice (6-8 week) were ordered from Shanghai SLAC Laboratory Animal Co., Ltd. All mice were housed at Zhejiang University Laboratory Animal Center in a Specific Pathogen Free (SPF) facility with individually ventilated cages. The room has controlled temperature (20-22°C), humidity (30%-70%), and light (12-hour light-dark cycle). Mice were provided ad libitum access to a regular rodent chow diet. The testes were collected and washed in ice-cold DPBS. The testes were minced and digested using 1 mg/ml collagenase II (Sigma, C6885) for 20 min at 37°C under slow rotation. After digestion, the cell suspension was slowly filtered through a

40-μm cell strainer (Biologix, 151040) to obtain single cells. Cells were washed and resuspended in cold DPBS.

Fabrication of the microwell device

The hole diameter, depth, and distance of the Microwell-seq 2.0 were 50 μm, 60 μm, and 20 μm, respectively. The silicon and polydimethylsiloxane (PDMS) plates (18 mm x 18 mm) were manufactured by ZhongXinQiHeng (Suzhou). Both the silicon and PDMS plates are reusable. Disposable agarose microwell plates were made by pouring 5% agarose solution onto the surface of the PDMS plates[1]. The agarose microwell plates can be stored using clean and sterile DPBS at 4°C for up to one month.

Synthesis of barcoded beads

Magnetic beads (45 μm in diameter) coated with carboxyl groups were provided by Suzhou Knowledge & Benefit Sphere Tech. Co., Ltd. (Suzhou). The barcoded oligonucleotides on the surface of the beads were synthesized by three rounds of split-pool as those reported previously[1]. All oligonucleotide sequences (v2_Bead_A,

v2_Bead_B, and v2_Bead_C) are provided in Supplementary Table S7. All oligonucleotides were synthesized by Sangon Biotech Co., Ltd. with high-performance liquid chromatography purification.

Cell fixation

Cells were fixed using methanol as previously reported[2]. Cells were collected and washed twice in ice-cold PBS. 5 x 10^6 cells were resuspended in 1 mL inPBS (1% RNase Inhibitor (Vazyme, R301), 1% 20 mg/ml BSA (Sangon, B600036), and 1% 10 mM DTT (Sangon, A620058)). 4 mL chilled 100% methanol was added drop by drop. Cells were stirred gently with the pipette tip and incubated for 30 min at −20°C. Fixed cells can be stored at −80°C for up to one month. After fixation, cells were pelleted at 500 x g for 5 min (4°C) and washed twice with ice-cold inPBS. Cells were resuspended in ice-cold inPBS and filtered through a 40-μm cell strainer. Filtered cells were counted and diluted for reverse transcription.

Reverse transcription

RT primers (100 μM) and RT oligo bridge (100 μM) were annealed 1:1 in 96 well plates by heating to 95°C for 2 min and cooling down to 20°C at a rate of -0.1°C per second. For each well of the 96 well plate, 5 x 10^4 cells in 5.5 μL inPBS, 0.5 μL 10 mM dNTP, and 1 μL annealed RT primer (50 μM) were added and mixed gently. The plate was incubated at 55°C for 5 min and incubated on ice immediately. Prepare the reverse transcription reaction mix (for 110 wells): 220 μL 5 x RT Buffer, 55 μL reverse transcriptase, 55 μL RNase Inhibitor. RT reaction mixture was distributed to each well (3 μL/well) and stirred gently with the pipette tip.

Two thermal cyclings were tested. RT-cycling-42°C: (8°C for 12 s, 15°C for 45 s, 20°C for 45 s, 30°C for 30 s, 42°C for 2 min) x 3 cycles, 42°C for 45 min; RT-cycling-55°C: (8°C for 12 s, 15°C for 45 s, 20°C for 45 s, 30°C for 30 s, 42°C for 2 min, and 55°C for 3 min) x 3 cycles, 55°C for 45 min. After RT, Microwell-seq 1.0 lysis buffer[1] was added and cDNA was enriched using 1 x VAHTS DNA Clean Beads (Vazyme, N411). RT efficiency was compared using TaqMan qPCR (AceQ qPCR Probe Master Mix, Vazyme, Q112).

Three reverse transcriptases were tested using RT-cycling-42, including Hiscript III Reverse Transcriptase (Vazyme, R302), PrimeScript II Reverse Transcriptase (Takara, 2690A), and Maxima H Minus (Thermo, EP0751). RT efficiency was compared using TaqMan qPCR.

RT time was also tested. Cells were collected and lysed at three time points: 30 min, 60 min, and 90 min. RT efficiency was compared using TaqMan qPCR.

RT-plate movement modes (rotative and static) were tested. The first 3 cycles of RT cycling were performed in PCR machine. The plate was turned upside down every 10 min. After 3 cycles, the rotative mode plate was transferred into an incubator with slow rotation; the static mode plate was incubated in PCR machine and turned upside down every 10 min. RT efficiency was compared using TaqMan qPCR.

Four RT primers were tested, including: v2_RT_25T (poly-T, barcode 1-32), v2_RT_15T (poly-T, barcode 33-64), v2_RT_25T_VN (poly-T, barcode 65-96) and v2_RT_6N. RT efficiency was compared using TaqMan qPCR and NGS. All oligonucleotide sequences (v2_RT_25T, v2_RT_15T, v2_RT_6N, v2_RT_25T_VN, and v2_RT_Bridge) are provided in Supplementary Table S8.

RT buffers and additive betaine were tested. 5 x RT buffer (Maxima, 250 mM Tris-HCl (pH 8.3 at 25°C); 375 mM KCl; 15 mM $MgCl_2$; 50 mM DTT) and 5 x NaCl buffer (250 mM Tris-HCl (pH 8.3 at 25°C); 375 mM NaCl; 15 mM $MgCl_2$; 50 mM DTT)[3]. 1M betaine (Sigma) was also tested in RT reaction. RT efficiency was compared using TaqMan qPCR and NGS.

Cell and bead loading

Compared with the Microwell-seq 1.0-plate, Microwell-seq 2.0-plate was designed with a honeycomb-like arrangement. Both beads and plates were washed twice with ice-cold 3 x SSC. After RT, cells were collected and washed twice using ice-cold inPBS. Cells were resuspended in ice-cold inPBS and loaded on plate. Centrifugation was used to load cells. Press the “short” button for short centrifugation. Extra cells were washed away using ice-cold PBS gently. Bead suspension was then loaded on the microwell plate, which was placed on a magnet. Extra beads were washed away using ice-cold 3 x SSC. Beads with different sizes were tested (28 μm and 45 μm).

Cell lysis and hybridization

200 μL lysis buffer was added to lysis cells and removed after 30 min of incubation at room temperature. Based on Microwell-seq 1.0 lysis buffer (0.1 M Tris-HCl pH 7.5, 0.5 M LiCl, 1% SDS, 10 mM EDTA, 5 mM DTT), 2.0 lysis buffer was designed (50% Formamide, 5 x SSC, 1% SDS, 10 mM EDTA). The proportion of Formamide (10%, 20%, 50%), SSC (2x, 5x), additive PEG 8000 (0%, 2%, 4%, 10%) and T4-lysis buffer (1% SDS, 1 x T4 buffer, 10 mM EDTA, ± 10% PEG 8000) were also tested. Hybridization efficiency was compared using TaqMan qPCR and NGS.

Ligation

Beads were collected and transferred to an RNase-free tube, washed once with 1 mL of 6 x SSC, once with 500 μL of 6 x SSC and then once with 200 μL of 50 mM Tris-HCl pH 8.0. Beads were resuspended in ligation solution. Three ligases were tested, including Ampligase (Lucigen, A0102K), T4 Ligase (Thermo, EL0012), and E. coli Ligase (NEB, M0205). Ampligase (Ampligase 5 μL, Sulfolobus DNA Polymerase IV (NEB, M0327S) 1 μL, Buffer 5 μL, RNase inhibitor 1 μL, ddH_2O 36 μL, dNTP 2 μL,

37°C, rotation, 1h); T4 Ligase (T4 ligase 1 μL, Buffer 5 μL, RNase inhibitor 1 μL, ddH_2O 41 μL, dNTP 2 μL, 16°C, rotation, 1h); E. coli Ligase (E. coli Ligase 1μL, Sulfolobus DNA Polymerase IV 1 μL, Buffer 5 μL, RNase inhibitor 1 μL, ddH_2O 40 μL, dNTP 2 μL, 16°C, rotation, 1h). Efficiency was compared using TaqMan qPCR.

Exonuclease digestion

After ligation, beads were washed with 200 μL of TE-SDS, 200 μL of TE-TW and 200 μL of 10 mM Tris-HCl (pH 8.0). Beads were suspended in 200 μL of exonuclease I mix (5 μL exonuclease I (NEB, M0293L), 20 μL buffer, 1μL RNase Inhibitor, 174 μL ddH_2O), and incubated at 37°C (rotative mode) for 60 min to remove oligonucleotides that did not capture cDNA/mRNA.

Second-strand synthesis

Beads were pooled and washed once with 200 μL of TE-SDS, twice with 200 μL of TE-TW, and resuspended in 500 μL 0.1 M NaOH. The tube was rotated for 5 min at room temperature. Beads were washed twice with 500 μL of TE-TW, and once with

500 μL 10 mM Tris-HCl pH 8.0. Two 2nd strand synthesis strategies were tested. Primer plus[4]: Beads were resuspended in 200 μL 2nd strand synthesis reaction (5 μL Klenow fragment exo- (Vazyme, N105-01), 40 μL Maxima 5 x RT Buffer, 80 μL 30% PEG8000, 20 μL 10 mM dNTPs, 20 μL 100 μM dN-TSO primer, 35 μL ddH_2O) and rotated end-over-end at 37°C for 60 min; Primer minus: Beads were resuspended in primer reaction (20 μL dN-TSO primer, 10 mM Tris-HCl pH 8.0, 50 mM NaCl, 1 mM EDTA) and rotated end-over-end at 37°C for 20 min, and then primer reaction was replaced by 2nd strand synthesis reaction (5 μL Klenow fragment exo-, 40 μL Maxima 5 x RT Buffer, 80 μL 30% PEG8000, 20 μL 10 mM dNTPs, 55 μL ddH_2O). Buffers were also tested (Maxima 5 x RT Buffer and Klenow fragment exo- 10 x Buffer). Efficiency was compared using TaqMan qPCR and NGS.

cDNA PCR

After 2nd strand synthesis, beads were washed once with 200 μL of TE-SDS, twice with 200 μL of TE-TW, and once with 500 μL 10 mM Tris-HCl pH 8.0. Beads were resuspended in PCR reaction (50 μL 2 x KAPA HiFi HotStart Ready Mix (KAPA,

KK2602), 2 μL 10 μM TSO-PCR primer, 48 μL ddH_2O). The PCR program was as follows: 98°C for 3 min; 6 cycles of 98°C 20 s, 65°C 45 s, and 72°C 6 min; 6 cycles of 98°C 20 s, 67°C 20 s, and 72°C 6 min; 72°C 10 min and 4°C hold. PCR products were purified using 0.8 x VAHTS DNA Clean Beads.

Library preparation

The purified cDNA library was fragmented by a customized transposase that carries two identical insertion sequences. The customized transposase was from TruePrep DNA Library Prep Kit V2 for Illumina (Vazyme, TD502). The fragmentation reaction was performed following the instructions of the manufacturer. We replaced the index 2 primers (N5XX/N7XX) in the kit with MGI-P5s primer and MGI-P7 primers (Supplementary Table S9) to specifically amplify fragments that contain the 3' end of transcripts. The PCR program was as follows: 72°C 3 min; 98°C 30 s; 14 cycles of 98°C 15 s, 60°C 30 s, and 72°C 3 min; 72°C 5 min and 4°C hold. To eliminate primer-dimers and large fragments, VAHTS DNA Clean Beads (R1=0.6 x, R2=0.15 x) were used to purify the cDNA library. Then, size distribution was analyzed on an Agilent

2100 bioanalyzer, and a peak at approximately 300-500 bp range should be observed. Circularization was performed to obtain a sequencing nanoball library for MGI DNBSEQ-T7 using VAHTS Circularization Kit for MGI (Vazyme, NM201).

Microwell-2.0-ATAC-seq

5 x 10^6 mixed cells (293T and 3T3) were resuspended in 1 mL ice-cold lysis buffer (0.1% IGEPAL CA-630 (Sigma), 0.01% digitonin (Thermo), 0.1% Tween-20 (Diamond), 1% BSA, 10 mM Tris-HCL pH 7.5, 10 mM NaCl (Sangon), 3 mM $MgCl_2$ (Thermo) in ddH_2O). The lysis was performed on ice for 3 min, and 5 mL of RSBT (0.1% Tween-20, 1% BSA, 10 mM Tris-HCL pH 7.5, 10 mM NaCl, 3 mM $MgCl_2$ in in ddH_2O) was added, and the medium was then filtered through a 40-μm strainer to remove the debris and clumps. Nuclei were washed with RSBT once and resuspended with 5 mL PBS. 140 μL 37% formaldehyde solution (HUSHI) was added for a final concentration at 1%. Nuclei were incubated at room temperature for 10 min. Then 250 μL 2.5 M Glycine (Diamond) was added to quench the reaction at room temperature for 5min, and finally the mixture was placed on ice for 15 min to stop cross-linking

completely. Prepare the freezing buffer (50 mM Tris-HCL pH 8.0, 25% Glycerol (Sangon), 5 mM $Mg(OAc)_2$ (Sangon), 0.1 mM EDTA in ddH_2O). Fixed nuclei were washed with 1 mL RSBT, and the pellet could be resuspended in 1 mL freezing solution (975 μL freezing buffer, 5 μL 5mM DTT, 20 μL 50 x protease inhibitor cocktail) and stored at -80°C.

96 uniquely indexed Tn5 transposomes were assembled as follow. For each well of the 96-well plate, the Tn5 primers (25 pM Tn5_primer_A, 50 pM TN5_ME, 25 pM Barcoded_Tn5_primer_B, all oligonucleotide sequences are provided in Supplementary Table S8) were mixed. The plate was incubated at 95°C for 2 min, and cooled down to 20°C at a rate of -0.1°C per second. A high concentration of Tn5 (Vazyme) was diluted and mixed with annealed Tn5 primers, and the mixture was then incubated at 30°C for 60 min. The final indexed Tn5 concentration was 40 ng/μLL. Frozen nuclei in freezing solution were thawed at 37°C and centrifuged at 500 g for 5 min. Nuclei were then resuspended with 1 mL RSBT and filtered through a 40-μm strainer to remove clumps. After filtering, the nuclei were centrifuged at 500 g for 5 min. Nuclei were resuspended with tagmentation buffer (10 mM Tris-HCl pH 7.5, 5

mM $MgCl_2$, 10% DMF (Sigma), 1% BSA, 0.1% Tween-20, 0.01% digitonin and 0.4 x PBS). After counting, nuclei were then splitted into a 96-well plate. For each well, 23.5 μL nuclei (10k) were mixed with 1.5 μL indexed Tn5 gently. Then the tagmentation was carried out at 55°C for 30 min, and 25 μL 2 x Stop Buffer (25 mL 40 mM EDTA, 3.9 μL 6.4 M Spermidine) was added into each well. The plate was incubated at 37°C for 15 min to completely stop the tagmentation. Then all the nuclei were pooled together into a 1.5 mL centrifuge tube and centrifuged at 500 g for 5 min. Nuclei were washed with RSBT, and resuspended with 40 μL RSBT. 60 μL PNK mix (10 μL 10 x PNK buffer (NEB), 20 μL T4 Polynucleotide Kinase (NEB), 10 μL 10mM ATP (NEB), 20 μL ddH_2O) was then added and the mixture was rotated for 5 times. Nuclei were incubated at 37°C for 30 min. After PNK reaction, nuclei were washed with RSBT and filtered using a 40 μm-strainer. After counting, 5 x 10^5 nuclei were resuspended in RSBT and loaded on plate. Centrifugation was used to load nuclei. 50 μL beads (for one plate) were mixed with 20 μL oligo bridge and annealed by heating to 95°C for 2 min and cooling down to 20°C at a rate of -0.1°C per second. Beads were resuspended in 3 x SSC and loaded on plate as Microwell-seq 2.0. 200 μL lysis buffer (20 μL 10%

SDS, 8 μL Proteinase K (Sangon), 20 μL 10 x T4 buffer, 40 μL 50% PEG 8000, 112 μL 10 mM Tris-HCl pH 8.0) was added to lysis nuclei and removed after 30 min of incubation at room temperature. Beads were collected and transferred to an RNase-free tube, washed once with 1 mL of 6 x SSC, once with 500 μL of 6 x SSC and then once with 200 μL of 50 mM Tris-HCl pH 8.0. Beads were resuspended in ligation solution (T4 ligase 2 μL, Buffer 5 μL, ddH_2O 31 μL, dNTP 2 μL, 50% PEG 10 μL, 25°C, rotation, 1.5h). After ligation, beads were washed with 200 μL of TE-SDS, 200 μL of TE-TW and 200 μL of 10 mM Tris-HCl (pH 8.0). Beads were suspended in 200 μL of extension mix (5 x RT buffer 20 μL, dNTP 10 μL, Klenow fragment exo- 2.5 μL, 47.5 μL ddH_2O, 20 μL 50% PEG 8000), and incubated at 37°C for 60 min. Beads were washed once with 200 μL of TE-SDS, twice with 200 μL of TE-TW, and resuspended in 500 μL 0.1 M NaOH. The tube was rotated for 5 min at room temperature. Beads were washed twice with 500 μL of TE-TW, and once with 500 μL 10 mM Tris-HCl pH 8.0. Beads were suspended in 50 μL of PCR mix (25 μL KAPA, 1 μL 10 uM P5, 1 μL 10 uM P7, 23 μL ddH_2O). The PCR program was as follows: 98°C for 3 min; 2 cycles of 98°C 15 s, 60°C 30 s, and 72°C 1 min; 72°C 5 min, 98°C 3 min and 10°C hold immediately.

PCR product was collected and new PCR mix (19 μL KAPA, 1 μL 10 uM MGI 2100 P7/P5 primer mix) was added. The PCR program was as follows: 98°C for 3 min; 11 cycles of 98°C 15 s, 60°C 30 s, and 72°C 1 min; 72°C 5 min and 10°C hold. PCR product was purified with VAHTS DNA Clean Beads (R1=0.6 x, R2=0.15 x) for DNB sequencing.

TaqMan qPCR analysis

After RT, cells were lysed using Microwell-seq 1.0 lysis buffer and cDNA was purified using 1 x VAHTS DNA Clean Beads. The diluted cDNA was used as temples in TaqMan qPCR using AceQ qPCR Probe Master Mix (Vazyme, Q112). The TaqMan qPCR was performed with the Heal Force X960 Real-Time PCR. The qPCR program was as follows: 95°C 5 min; 40 cycles of 95°C 10 s, 60°C 30 s. 30-CT (Cycle Threshold) was compared. All oligonucleotide sequences are provided in Supplementary Table S10.

Compound preparation

The compounds were purchased from Targetmol and diluted in their respective vehicle (DMSO or ethanol) to 1000 x of their desired treatment concentration (Supplementary Table S3). The 16 small molecules are widely used to target the key pathways in stem cell biology, including TGFβ (LDN-193189, Repsox, SB431542), WNT (CHIR-99021, XAV-939, IWP-2), Hedgehog (Purmorphamine, SAG hydrochloride, Cyclopamine), FGF (PD0325901, PD173074), Notch (DAPT, LY411575), cAMP (Forskolin, HA-100), Retinoid Pathway Activators (Retinoic acid). The stock solutions were stored at -80°C until use.

Drug treatment

H9 human embryonic stem cells (ESCs) were maintained in mTeSR™1 media (STEMCELL Technologies) on 6-well tissue culture plates coated with Matrigel (BD Bioscience) routinely. Day 0, 96-well plate was coated using 50 µL Matrigel (25 µg protein/well). The 96-well plate was incubated at room temperature for 1 hour. H9 cells were dissociated into single-cell suspension with Accutase (Gibco) and resuspended in 1 mL mTeSR™1 (with 10 µg Y-27632) for counting. Excess Matrigel solution was

removed from the well and 5000 cells (200 μL) were seeded per well using a multi-channel pipettor. Cells were pipetted up and down several times with the multi-channel pipettor. After mixing, the plate was undisturbed inside the bench for 10 min. Then the plate was incubated at 37°C and 5% CO2 with 95% humidity overnight. Day 1, culture medium was removed and 100 μL fresh mTeSR™1 (with 25 μg protein/well Matrigel) was added. The plate was incubated at 37°C and 5% CO_2 with 95% humidity overnight. Day 2, a full medium change was performed using 100 μL fresh mTeSR™1. Day 3, a full medium change was performed using 200 μL fresh E6 (STEMCELL Technologies) with different compounds (47 perturbations + 1 vehicle, two repetitions) (Supplementary Table S4). Day 5, cells were dissociated into single-cell suspension with Accutase. Cells were collected into a 96-well PCR plate for washing and fixation. The fixed cells were stored at -80°C.

Processing of Microwell-seq 2.0 data

Microwell-seq 2.0 datasets were processed following previous protocols[1]. Reads were aligned to the *Homo sapiens* GRCh38 genome using STAR[5]. For mix-species

experiment, scRNA-seq reads were aligned to the mix genome (*Homo sapiens* GRCH39, *Mus musculus* GRCm38.88) assembly using STAR with default configurations. The DGE data matrices were obtained using the modified Drop-seq tools (https://github.com/ggjlab/mca_data_analysis/tree/master/preprocessing/Drop-seq_tools-1.12/) and the corresponding protocol is available at http://mccarrolllab.org/dropseq/. The DGE data containing the top 50,000 cells sorted by the total number of transcriptions were obtained after this pre-procession.

For quality control, we filtered out cells with the detection of fewer than 300 transcripts. Cells with a high proportion of transcript counts derived from mitochondria-encoded genes were also excluded.

Pre-processing of Microwell-2.0-ATAC-seq data

For sequenced Microwell-2.0-ATAC-seq library, we integrated the concatenated barcode at the beginning of the read name in the demultiplexed FASTQ using Drop-seq tools. Then, snaptools[6] was used to pre-process the Microwell-2.0-ATAC-seq datasets. Demultiplexed sequencing reads are aligned to the merged hg19-mm10

genome reference (provided by Drop-seq group, GSE63269) using bwa-mem[7] in pair-end mode with default parameter settings. For cell quality control, the single nucleus accessibility profile containing the top 10,000 cells sorted by the total number of unique mapping reads were obtained after this pre-procession. The corresponding protocol is available at https://github.com/r3fang/SnapTools.

The fragment size distribution was evaluated using insertion size of the aligned bam. TSS enrichment were calculated using deeptools[8] bamCoverage and computeMatrix command with the corresponding reference. Nonduplicate fragment endpoints for each cell were used for peak calling in each sample by use of MACS2[9] callpeak command with parameters "–shift -100 –extsize 200 –nomodel". Fraction of reads in peaks (FRiP) were then calculated using bedtools[10] intersect command. ChIPseeker[11] was applied to assign peaks to nearest or overlapping gene, exon, intron, promoter, 5′ untranslated region (UTR), 3′ UTR, and other genomic features.

To compare with bulk ATAC-seq result, we downloaded and pre-process bulk ATAC-seq data of the corresponding 3T3 (GSM3383675) cell line using the same pipeline. We compared the signal tracks generated from BAM file in chromosome 8: 0-

250,000,000 region of mouse reference. Then, the peak overlap was calculated using ChIPpeakAnno package.

Analysis of Microwell-seq 2.0 data

Seurat[12] was used as a tool for cell clustering on a per-dataset basis. The data were log_2(counts per million (CPM)/1000+1)-transformed, and the number of UMIs and the percentage of mitochondrial gene content were regressed out. A total of 2,000 genes were selected through the 'FindVariableFeatures' function by using the 'vst' method as inputs for initial principal component analysis (PCA) and the number of principal components (PCs) used for nonlinear dimensional reduction (*t*-SNE). For clustering, we set different resolution parameters between 0.1 and 0.3 in the "FindAllCluster" function and narrowed down to certain cluster numbers by distinguishing differential genes among clusters. We use the 'FindAllMarkers' function to find out the markers among the clusters or different treatments and show them by using the function 'DoHeatmap'. These parameters, including the resolution and number of PCs, were adjusted on a per-dataset basis. The testis was annotated with marker genes reported

previously[13]. As the 48 different treatments identified by the RT barcode, we annotated cells as their treatments by spliting the RT barcode from cell barcode. To identify different treatments causing which disturbing trend, we gave every treatment a cluster label which occupied the highest proportion in the treatment. Scripts for processing Microwell-seq 2.0 were available at https://github.com/ggjlab/MW2.

48 different treatments are represented by 96 RT-barcodes which can be identified by the last 10 bases in every cell barcode. PAGA[14] is used to infer the cell trajectory based on estimating connectivity of manifold partitions in the scanpy environment. First, we preprocessed the dataset by using recipe zheng[15] to find high variable genes, and using n_neighbors=10, n_pcs=20 after PCA. Then we computed a coarse-grained and simplified PAGA graph where each point represents a separate treatment. Based on the coarse-grained PAGA graph, we recomputed the embedding using PAGA-initialization and tagged cells with their treatment information when visualization by using the PAGA embedding graph. For diffusion pseudotime in the chosen cells, the root cell was set as a random cell in the treatment within the least drugs. The visualization in python is realized by the function sc.pl.draw_graph.

To identify correlated cell types between two cell atlas datasets[16], we first aggregated the cell-type-specific UMI counts, normalized by the total count, multiplied by 100,000 and log-transformed after adding a pseudocount. We then applied non-negative least-squares (NNLS) regression to predict the gene expression of target cell type (*Ta*) in dataset A with the gene expression of all cell types (*Mb*) in dataset B: $Ta = \beta 0a + \beta 1aMb$, in which *Ta* and *Mb* represent filtered gene expression for target cell type from dataset A and all cell types from dataset B, respectively. To improve accuracy and specificity, we selected cell-type-specific genes for each target cell type by (1) ranking genes on the basis of the expression fold-change between the target cell type versus the mean expression across all cell types, and then selecting the top 200 genes; (2) ranking genes on the basis of the expression fold-change between the target cell type versus the cell type with maximum expression among all other cell types, and then selecting the top 200 genes; and (3) merging the gene lists from steps (1) and (2). $\beta 1a$is the correlation coefficient computed by NNLS regression. $\beta 0a$ is the intersect of the regression line with the y axis when *Mb* is zero. Similarly, we then switch the order of datasets A and B, and predict the gene expression of target cell type (*Tb*) in dataset B with the gene

expression of all cell types (*Ma*) in dataset A: $Tb = \beta 0b + \beta 1bMa$. Thus, each cell type *a* in dataset A and each cell type *b* in dataset B are linked by two correlation coefficients from the above analysis: βab for predicting cell type *a* using *b*, and βba for predicting cell type *b* using *a*. We combine the two values by $\beta = 2(\beta ab + 0.01)(\beta ba + 0.01)$, and find that β reflects the matching of cell types between two datasets with high specificity.

Supplementary Figs

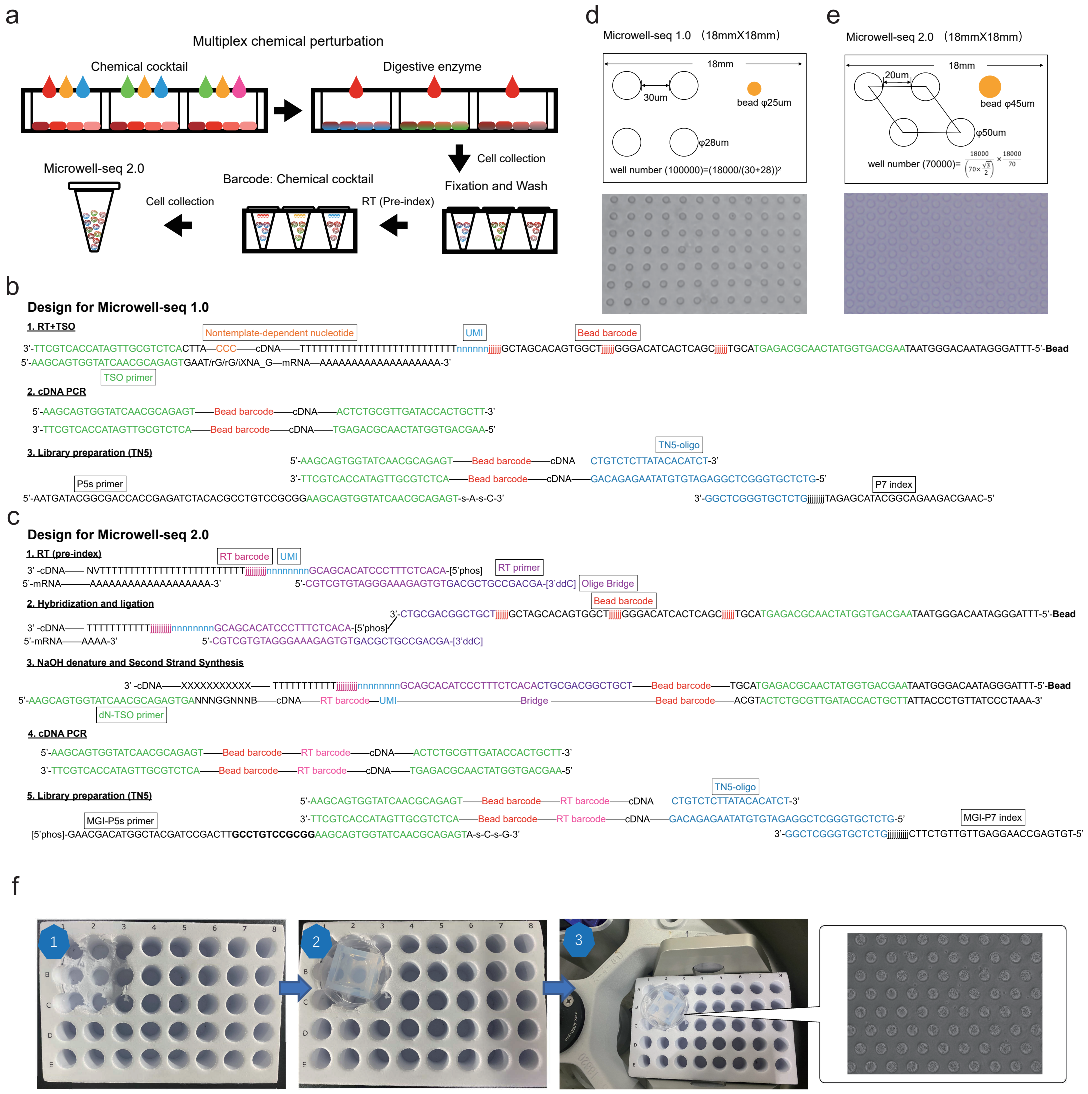

Supplementary Fig. S1 Microwell-seq 2.0 combines in-cell RT and microwell-based scRNA-seq. a Schematic of multiplexed chemical perturbation using Microwell-seq 2.0. **b-c** Different designs of Microwell-seq 1.0 and 2.0. **d-e** Different designs of plate for Microwell-seq 1.0 and 2.0. **f** Schematic of cell loading with a centrifuge in Microwell-seq 2.0.

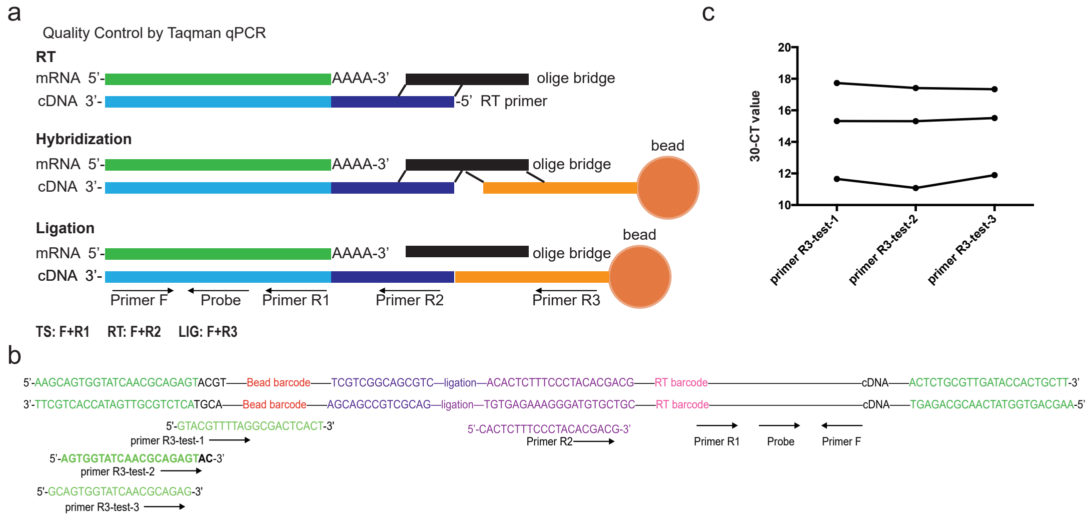

Supplementary Fig. S2 TaqMan qPCR-based optimization system. a-b Primer design for TaqMan qPCR. Three targets were selected: TS, Transcript; RT, Reverse transcription; LIG, Ligation. **c** Three LIG primers were checked with diluted cDNA.

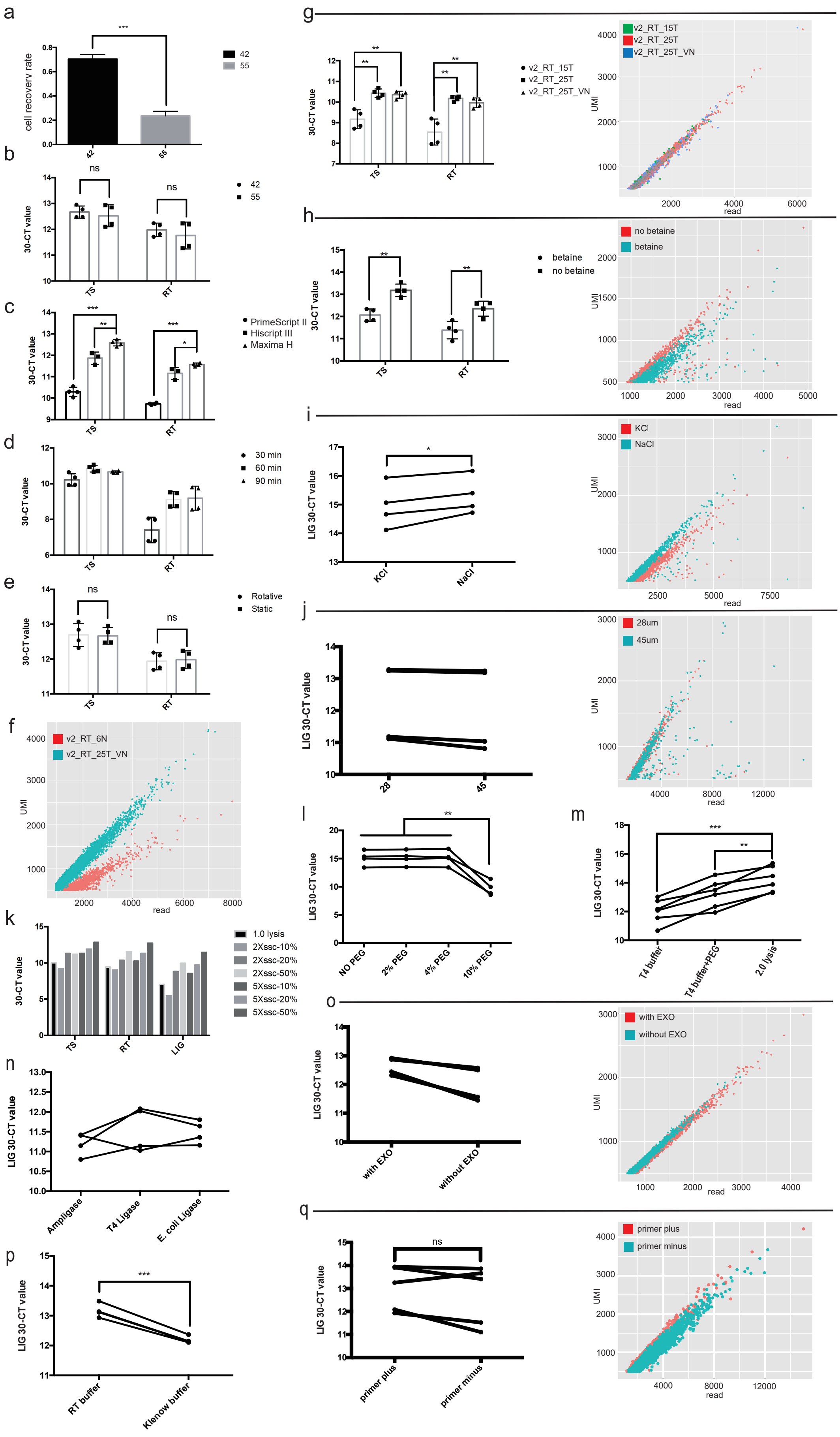

a
cell recovery rate
***
42
55
b
30-CT value
ns
ns
TS
RT
c
30-CT value
***
**
***
*
PrimeScript II
Hiscript III
Maxima H
d
30-CT value
30 min
60 min
90 min
e
30-CT value
Rotative
Static
f
v2_RT_6N
v2_RT_25T_VN
UMI
read
g
30-CT value
v2_RT_15T
v2_RT_25T
v2_RT_25T_VN
h
30-CT value
betaine
no betaine
i
LIG 30-CT value
KCl
NaCl
j
LIG 30-CT value
28
45
28um
45um
k
30-CT value
1.0 lysis
2Xssc-10%
2Xssc-20%
2Xssc-50%
5Xssc-10%
5Xssc-20%
5Xssc-50%
LIG
l
LIG 30-CT value
NO PEG
2% PEG
4% PEG
10% PEG
m
LIG 30-CT value
T4 buffer
T4 buffer+PEG
2.0 lysis
n
LIG 30-CT value
Ampligase
T4 Ligase
E. coli Ligase
o
LIG 30-CT value
with EXO
without EXO
p
LIG 30-CT value
RT buffer
Klenow buffer
q
LIG 30-CT value
primer plus
primer minus

Supplementary Fig. S3 Optimization of Microwell-seq 2.0 using TaqMan qPCR and NGS. a Cell recovery rate with different RT temperatures (RT-cycling-42°C and RT-cycling-55°C). data are means ± SD, n = 4; *P* values were calculated by Student's t-test. **b-i** qPCR and NGS analysis of different RT conditions. RT temperature (42°C and 55°C) (**b**); reverse transcriptase (PrimeScript II Reverse Transcriptase, Takara; Hiscript III Reverse Transcriptase, Vazyme; Maxima H Minus, Thermo) (**c**); RT time (30 min, 60 min, and 90 min) (**d**); plate movement mode (rotative and static) (**e**); RT primer (v2_RT_25T, v2_RT_15T, v2_RT_6N, and v2_RT_25T_VN) (**f, g**); RT additive betaine (**h**); RT buffer (KCl and NaCl) (**i**). **(b, c, d, e, g, h, i,** data are means ± SD, n ≥ 3; *P* values were calculated by Student's t-test). Scatter plots of NGS show the transcript number versus read number of each individual cell (**f, g, h, i**). **j** qPCR and NGS analysis of beads size in Microwell-seq 2.0. Scatter plot of NGS shows the transcript number versus read number of each individual cell. **k-m** qPCR analysis of lysis buffer in Microwell-seq 2.0. Bulk cell samples were mixed with beads in different lysis buffers (Microwell-seq 1.0 lysis buffer, 2.0 lysis buffer with 2 x – 5 x SSC, and 10%-50% Formamide) (**k**). 2.0 lysis buffer with 0%, 2%, 4%, 10% PEG 8000 were

used in Microwell-seq 2.0 (**l**). T4 buffer ±10%PEG 8000 versus 2.0 lysis buffer (**m**). (**l**, **m**, *P* values were calculated by Student's t-test, n ≥ 4). **n** qPCR analysis of ligation in Microwell-seq 2.0. Ampligase (Lucigen, A0102K), T4 Ligase (Thermo, EL0012), and E. coli Ligase (NEB, M0205) were used. **o** qPCR and NGS analysis of exonuclease digestion in Microwell-seq 2.0. Scatter plot of NGS shows the transcript number versus read number of each individual cell. **p-q** qPCR and NGS analysis of second-strand synthesis in Microwell-seq 2.0. Second-strand synthesis was performed with two different buffers (Maxima 5 x RT Buffer and Klenow Exo- 10 x Buffer) (**p**). Two second-strand synthesis strategies (primer plus and primer minus) were tested (**q**). (**p**, **q**, *P* values were calculated by Student's t-test, n ≥ 3). Scatter plot of NGS shows the transcript number versus read number of each individual cell (**q**). The summary of NGS in this figure was listed in Supplementary Table S6. ns: no significance, * p-value<0.05, ** p-value<0.01, *** p-value<0.001.

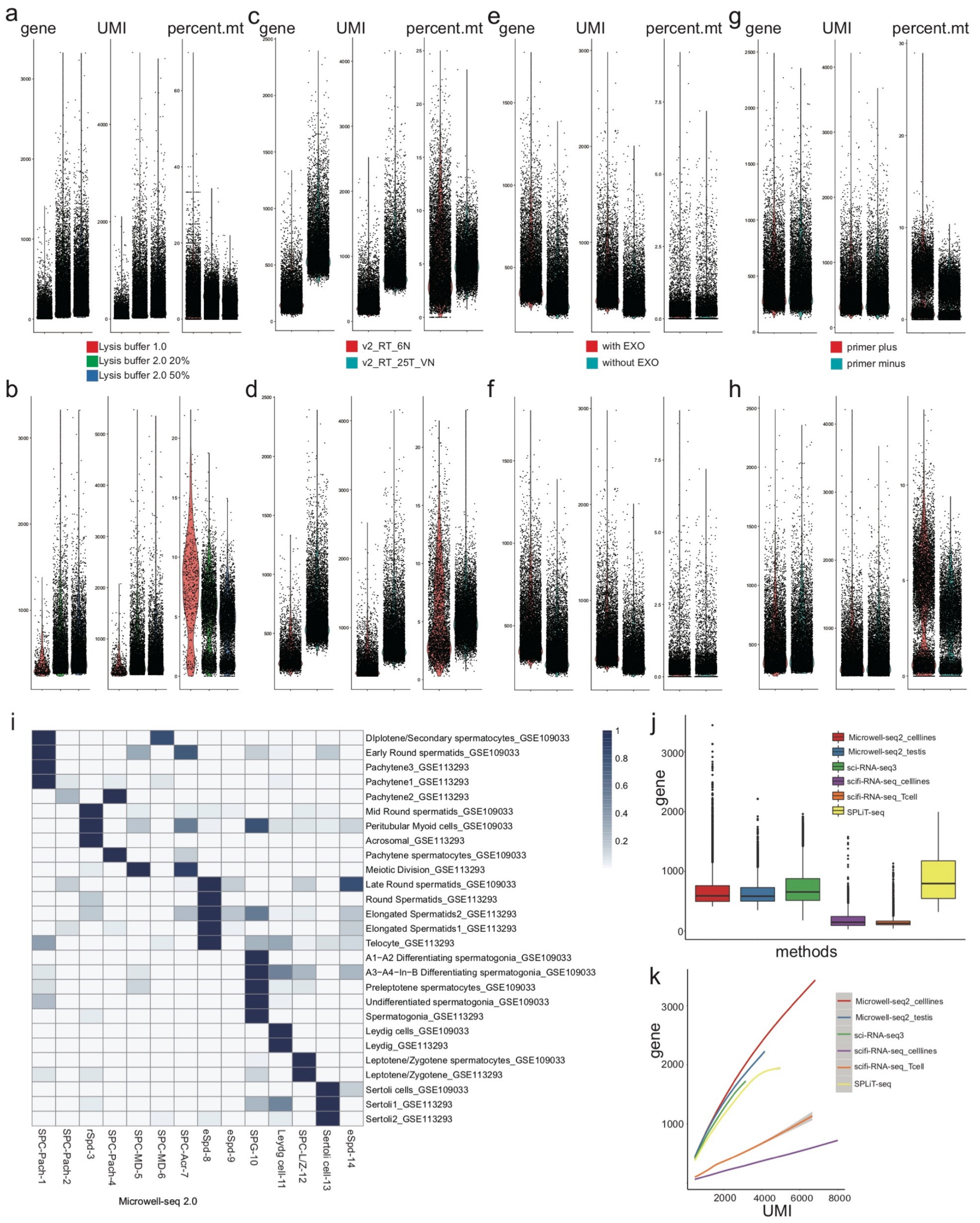

Supplementary Fig. S4 The quality control of Microwell-seq 2.0. a-h Gene, UMI, and precent.mt distribution plots. **a** and **b** for data in Fig. 1c; **c** and **d** for data in Fig. S3f; **e** and **f** for data in Fig. S3o; **g** and **h** for data in Fig. 3Sq. **a**, **c**, **e, g:** top 10000 cell; **b**, **d**, **f, h**: filter ≥ 300 UMI. **i** Cell-type correlation analysis matched cell types between

independently generated and annotated analyses of the adult mouse testis (Microwell-seq 2.0 (rows) versus GSE109033 and GSE113293 (columns)). All cell types identified by Microwell-seq 2.0 are shown. Colours correspond to beta values, normalized by the maximum beta value per row. **j-k** Number of genes detected per cell and UMI/gene plot for different methods with shallow-sequencing (UMI ≥ 500). Microwell-seq2 (GSM5395031); Microwell-seq2_testis (GSM5333088); sci-RNA-seq3 (GSE119945); scifi-RNA-seq_fourlines (GSM5151357); scifi-RNA-seq_Tcell (GSM5151353); SPLiT-seq (GSM3017261).

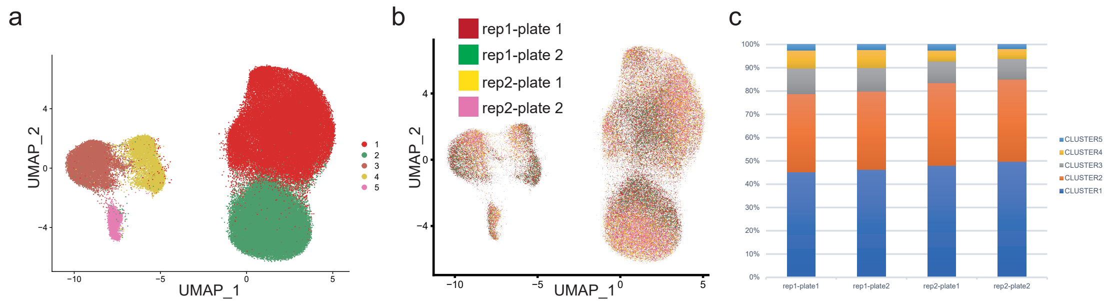

Supplementary Fig. S5 Resolving cellular heterogeneity in hESCs with multiplexed chemical perturbations. a-b UMAP analysis of 108 782 single cells sampled from hESCs with multiplexed chemical perturbations. Five cell-type clusters are labeled in the UMAP (**a**). Four batches are labeled in the UMAP (**b**). **c** The cell percentages of each cluster in four batches.

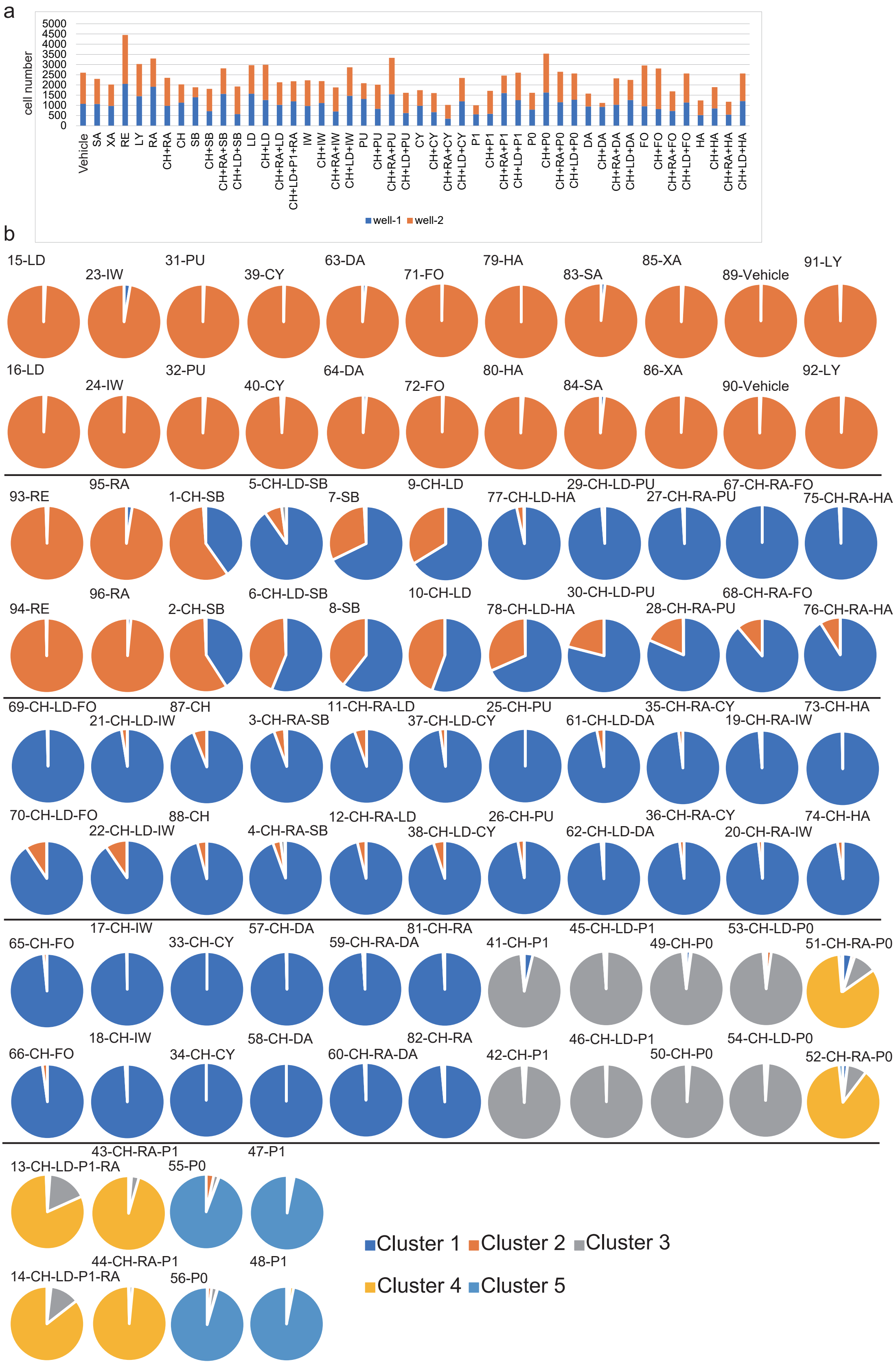
a
cell number
5000
4500
4000
3500
3000
2500
2000
1500
1000
500
0
Vehicle
SA
XA
RE
LY
RA
CH+RA
CH
SB
CH+SB
CH+RA+SB
CH+LD+SB
LD
CH+LD
CH+RA+LD
CH+LD+P1+RA
IW
CH+IW
CH+RA+IW
CH+LD+IW
PU
CH+PU
CH+RA+PU
CH+LD+PU
CY
CH+CY
CH+RA+CY
CH+LD+CY
P1
CH+P1
CH+RA+P1
CH+LD+P1
P0
CH+P0
CH+RA+P0
CH+LD+P0
DA
CH+DA
CH+RA+DA
CH+LD+DA
FO
CH+FO
CH+RA+FO
CH+LD+FO
HA
CH+HA
CH+RA+HA
CH+LD+HA
well-1
well-2
b
15-LD
23-IW
31-PU
39-CY
63-DA
71-FO
79-HA
83-SA
85-XA
89-Vehicle
91-LY
16-LD
24-IW
32-PU
40-CY
64-DA
72-FO
80-HA
84-SA
86-XA
90-Vehicle
92-LY
93-RE
95-RA
1-CH-SB
5-CH-LD-SB
7-SB
9-CH-LD
77-CH-LD-HA
29-CH-LD-PU
27-CH-RA-PU
67-CH-RA-FO
75-CH-RA-HA
94-RE
96-RA
2-CH-SB
6-CH-LD-SB
8-SB
10-CH-LD
78-CH-LD-HA
30-CH-LD-PU
28-CH-RA-PU
68-CH-RA-FO
76-CH-RA-HA
69-CH-LD-FO
21-CH-LD-IW
87-CH
3-CH-RA-SB
11-CH-RA-LD
37-CH-LD-CY
25-CH-PU
61-CH-LD-DA
35-CH-RA-CY
19-CH-RA-IW
73-CH-HA
70-CH-LD-FO
22-CH-LD-IW
88-CH
4-CH-RA-SB
12-CH-RA-LD
38-CH-LD-CY
26-CH-PU
62-CH-LD-DA
36-CH-RA-CY
20-CH-RA-IW
74-CH-HA
65-CH-FO
17-CH-IW
33-CH-CY
57-CH-DA
59-CH-RA-DA
81-CH-RA
41-CH-P1
45-CH-LD-P1
49-CH-P0
53-CH-LD-P0
51-CH-RA-P0
66-CH-FO
18-CH-IW
34-CH-CY
58-CH-DA
60-CH-RA-DA
82-CH-RA
42-CH-P1
46-CH-LD-P1
50-CH-P0
54-CH-LD-P0
52-CH-RA-P0
13-CH-LD-P1-RA
43-CH-RA-P1
55-P0
47-P1
14-CH-LD-P1-RA
44-CH-RA-P1
56-P0
48-P1
Cluster 1
Cluster 2
Cluster 3
Cluster 4
Cluster 5

Supplementary Fig. S6 Cell distribution of 48 small-molecule combinations. a Cell distribution of 48 small-molecule combinations with two repeats. **b** The cell percentages of each cluster in 48 combinations.

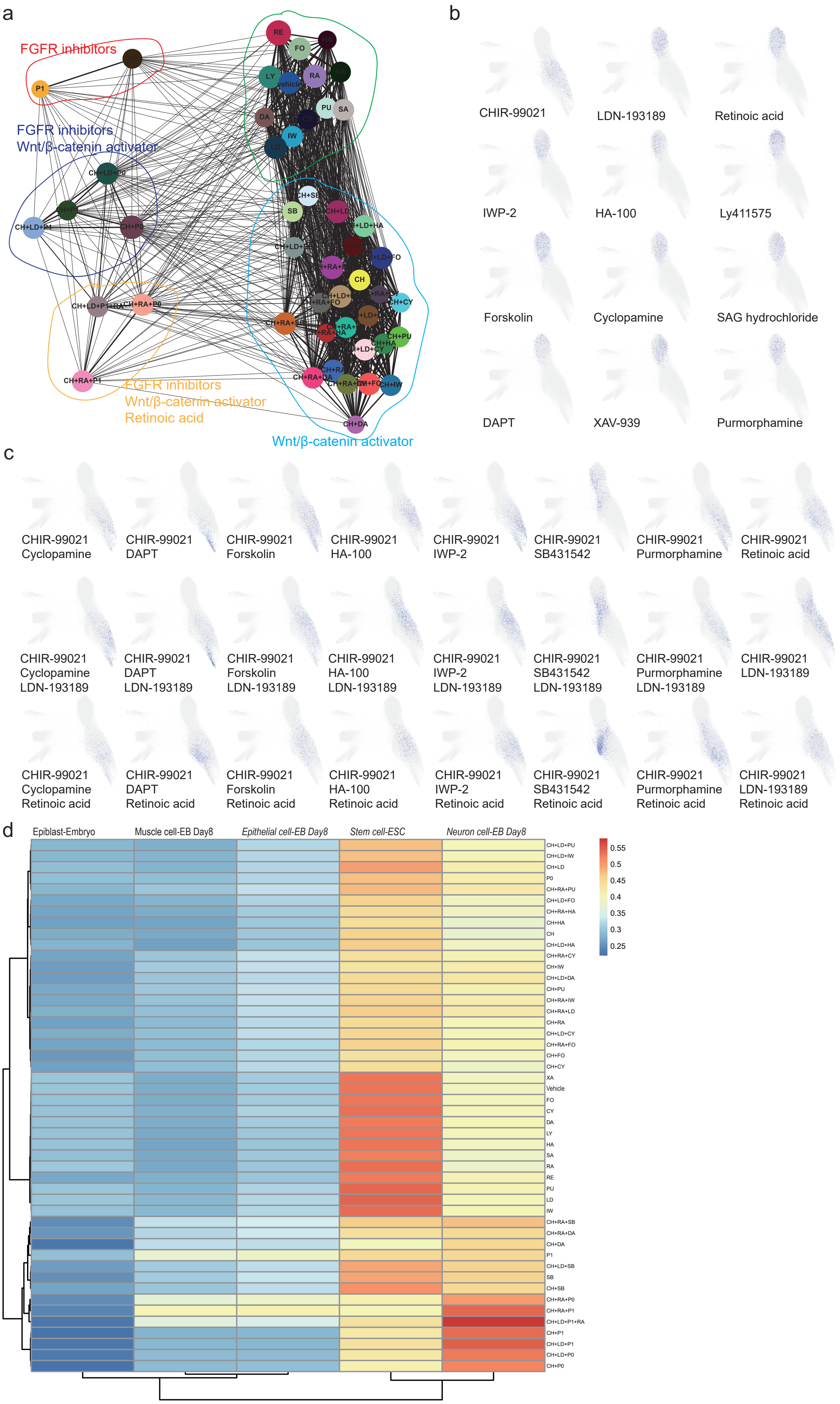
a
FGFR inhibitors
P1
FGFR inhibitors
Wnt/β-catenin activator
FGFR inhibitors
Wnt/β-catenin activator
Retinoic acid
Wnt/β-catenin activator
RE
FO
LY
RA
PU
SA
DA
IW
SB
CH
CH+DA
CH+RA+P1
b
CHIR-99021
LDN-193189
Retinoic acid
IWP-2
HA-100
Ly411575
Forskolin
Cyclopamine
SAG hydrochloride
DAPT
XAV-939
Purmorphamine
c
CHIR-99021 Cyclopamine
CHIR-99021 DAPT
CHIR-99021 Forskolin
CHIR-99021 HA-100
CHIR-99021 IWP-2
CHIR-99021 SB431542
CHIR-99021 Purmorphamine
CHIR-99021 Retinoic acid
CHIR-99021 Cyclopamine LDN-193189
CHIR-99021 DAPT LDN-193189
CHIR-99021 Forskolin LDN-193189
CHIR-99021 HA-100 LDN-193189
CHIR-99021 IWP-2 LDN-193189
CHIR-99021 SB431542 LDN-193189
CHIR-99021 Purmorphamine LDN-193189
CHIR-99021 LDN-193189
CHIR-99021 Cyclopamine Retinoic acid
CHIR-99021 DAPT Retinoic acid
CHIR-99021 Forskolin Retinoic acid
CHIR-99021 HA-100 Retinoic acid
CHIR-99021 IWP-2 Retinoic acid
CHIR-99021 SB431542 Retinoic acid
CHIR-99021 Purmorphamine Retinoic acid
CHIR-99021 LDN-193189 Retinoic acid
d
Epiblast-Embryo
Muscle cell-EB Day8
Epithelial cell-EB Day8
Stem cell-ESC
Neuron cell-EB Day8
CH+LD+PU
CH+LD+IW
CH+LD
P0
CH+RA+PU
CH+LD+FO
CH+RA+HA
CH+HA
CH
CH+LD+HA
CH+RA+CY
CH+IW
CH+LD+DA
CH+PU
CH+RA+IW
CH+RA+LD
CH+RA
CH+LD+CY
CH+RA+FO
CH+FO
CH+CY
XA
Vehicle
FO
CY
DA
LY
HA
SA
RA
RE
PU
LD
IW
CH+RA+SB
CH+RA+DA
CH+DA
P1
CH+LD+SB
SB
CH+SB
CH+RA+P0
CH+RA+P1
CH+LD+P1+RA
CH+P1
CH+LD+P1
CH+LD+P0
CH+P0
0.55
0.5
0.45
0.4
0.35
0.3
0.25

Supplementary Fig. S7 PAGA analysis of the cell transitions in chemical perturbation. a PAGA analysis of the chemical perturbation. Each node corresponds to a combination; node size represents the cell number. Thickness of the line represents the degree of connection. CHIR-99021 (CH) played an important role in the direction of branch 1; PD173074 (P1) and PD0325901 (P0) played an important role in the direction of branch 3,4, and 5. **b-c** PAGA plots show cell distribution of small-molecule combinations. **d** scHCL results for cells with 48 small-molecule combinations. Each row represents data from one small-molecule combinations; Each column represents cell type in HCL reference. Pearson correlation coefficient was used to evaluate cell-type gene expression similarity. Red indicates a high correlation; grey indicates a low correlation.

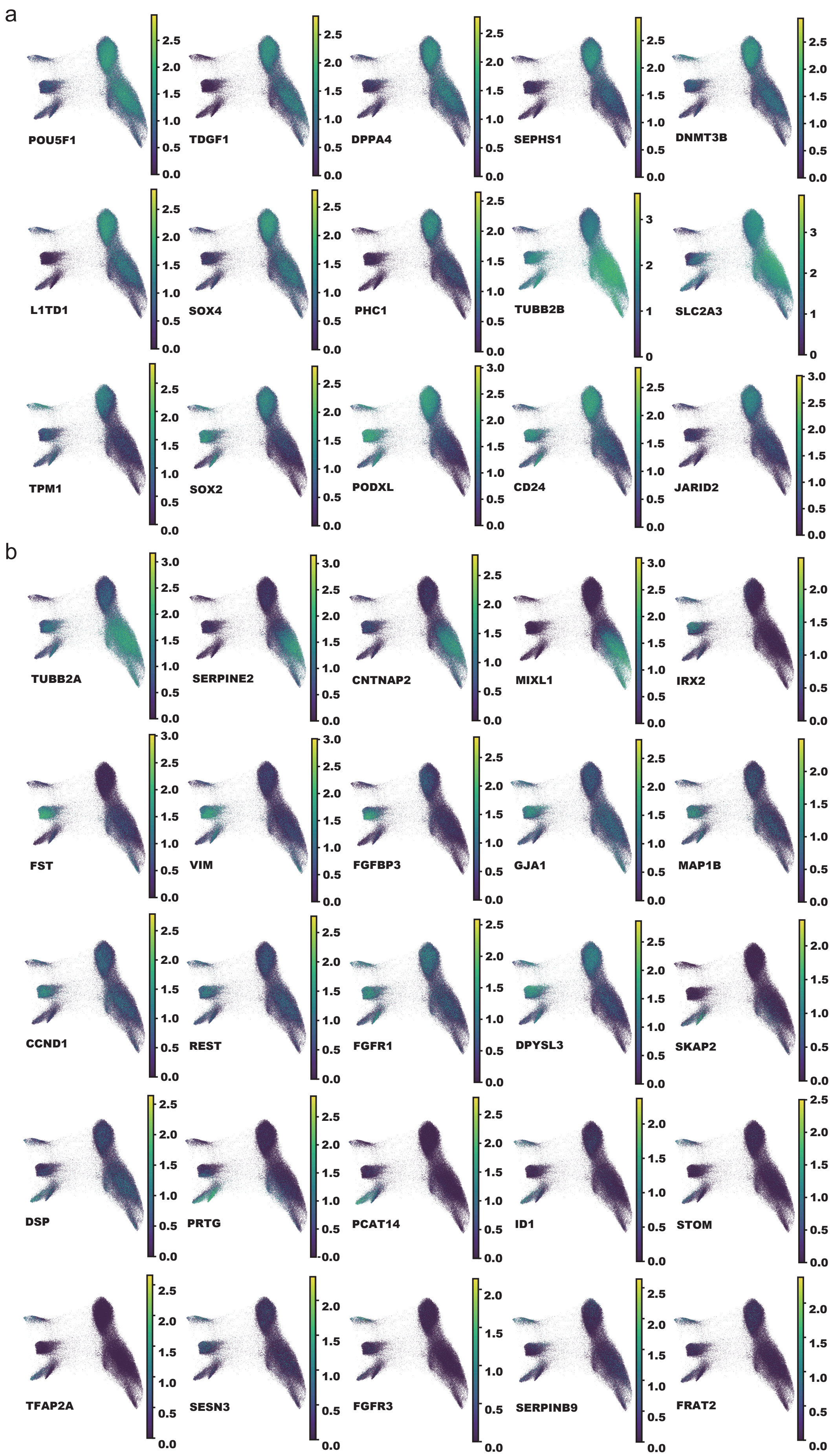

a
POU5F1
TDGF1
DPPA4
SEPHS1
DNMT3B
L1TD1
SOX4
PHC1
TUBB2B
SLC2A3
TPM1
SOX2
PODXL
CD24
JARID2
b
TUBB2A
SERPINE2
CNTNAP2
MIXL1
IRX2
FST
VIM
FGFBP3
GJA1
MAP1B
CCND1
REST
FGFR1
DPYSL3
SKAP2
DSP
PRTG
PCAT14
ID1
STOM
TFAP2A
SESN3
FGFR3
SERPINB9
FRAT2

Supplementary Fig. S8 Feature plots of PAGA show the branch-specific gene expression. Yellow corresponds to high expression levels; purple corresponds to low expression levels.

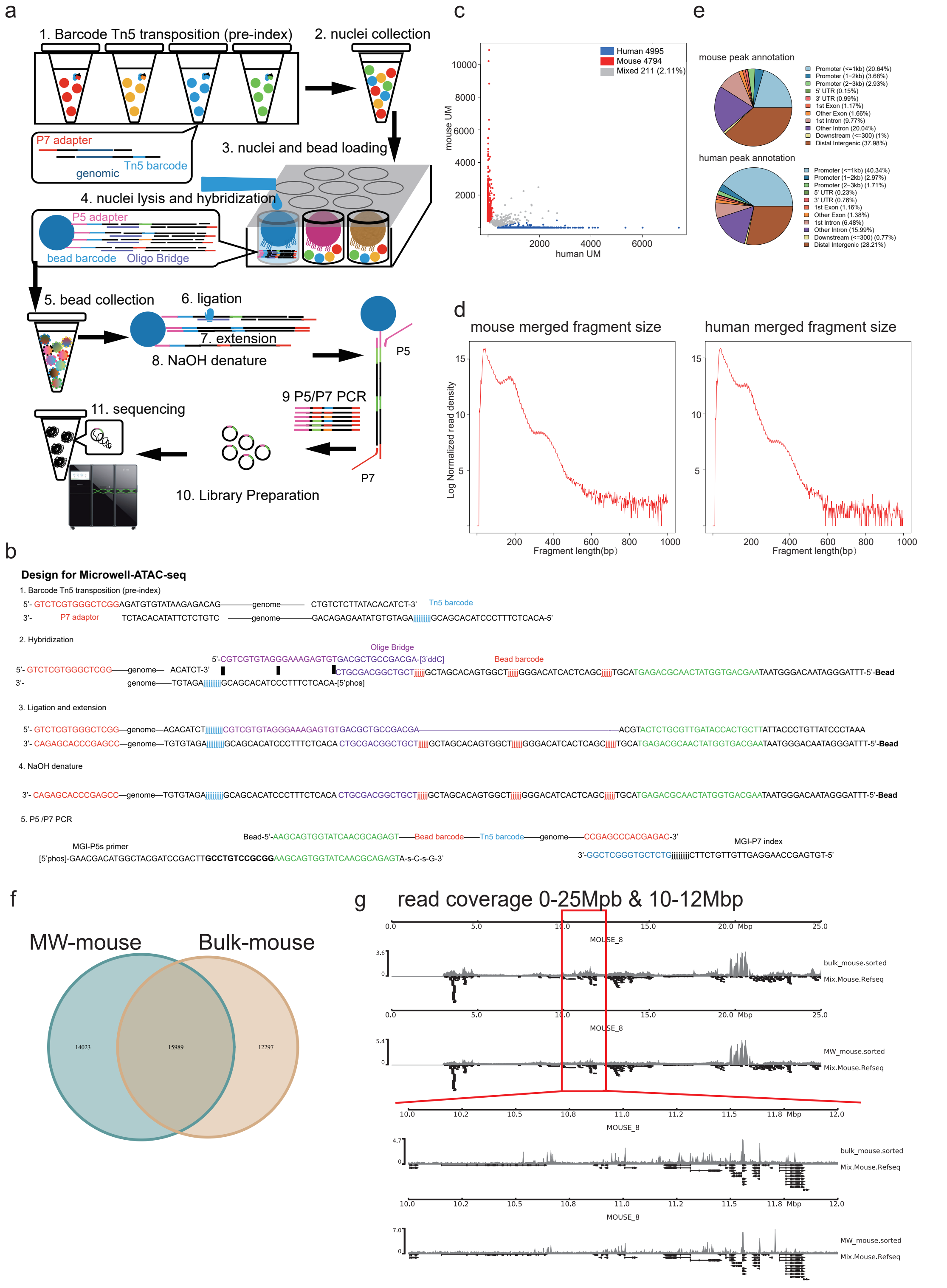

Supplementary Fig. S9 Microwell-2.0-ATAC-seq combines Microwell-seq and Tn5 transposition pre-index. a Schematic of Microwell-2.0-ATAC-seq. **b** Designs of

Microwell-2.0-ATAC-seq. **c** Scatter plot of human-mouse mix test using Microwell-2.0-ATAC-seq. The number of unique mapped reads aligning to the mouse or human genome from ATAC-seq profiles of human (293T) and mouse (3T3) cells by selecting the top 10000 cells according to the total number of unique mapping reads. Human (293T)/Mouse (3T3) cell mixing experiment showed a collision rate of 2.11%. **d** Fragment size distributions of Microwell-2.0-ATAC-seq data of Human (293T) and Mouse (3T3) chromatin, with a clear periodicity of approximately 200 bp. **e** Typical peak annotation pie charts of human and mouse. **f** The mouse peak overlap between bulk ATAC-seq and Microwell-2.0-ATAC-seq. **g** The comparison of the signal tracks generated from BAM file of bulk ATAC-seq and Microwell-2.0-ATAC-seq in chromosome 8: 0-250,000,000 region of mouse reference.

Supplementary Table S4 Array of drug treatment

96 well-RT barcode	1	2	3	4	5	6	7	8	9	10	11	12
A	1	9	17	25	33	41	49	57	65	73	81	89
B	2	10	18	26	34	42	50	58	66	74	82	90
C	3	11	19	27	35	43	51	59	67	75	83	91
D	4	12	20	28	36	44	52	60	68	76	84	92
E	5	13	21	29	37	45	53	61	69	77	85	93
F	6	14	22	30	38	46	54	62	70	78	86	94
G	7	15	23	31	39	47	55	63	71	79	87	95
H	8	16	24	32	40	48	56	64	72	80	88	96

96 well-chemical molecules	1	2	3	4	5	6	7	8	9	10	11	12
A	CH+SB	CH+LD	CH+IW	CH+PU	CH+CY	CH+P1	CH+P0	CH+DA	CH+FO	CH+HA	CH+RA	vehicle
B	CH+SB	CH+LD	CH+IW	CH+PU	CH+CY	CH+P1	CH+P0	CH+DA	CH+FO	CH+HA	CH+RA	vehicle
C	CH+RA+SB	CH+RA+LD	CH+RA+IW	CH+RA+PU	CH+RA+CY	CH+RA+P1	CH+RA+P0	CH+RA+DA	CH+RA+FO	CH+RA+HA	SA	LY
D	CH+RA+SB	CH+RA+LD	CH+RA+IW	CH+RA+PU	CH+RA+CY	CH+RA+P1	CH+RA+P0	CH+RA+DA	CH+RA+FO	CH+RA+HA	SA	LY
E	CH+LD+SB	CH+LD+P1+RA	CH+LD+IW	CH+LD+PU	CH+LD+CY	CH+LD+P1	CH+LD+P0	CH+LD+DA	CH+LD+FO	CH+LD+HA	XA	RE
F	CH+LD+SB	CH+LD+P1+RA	CH+LD+IW	CH+LD+PU	CH+LD+CY	CH+LD+P1	CH+LD+P0	CH+LD+DA	CH+LD+FO	CH+LD+HA	XA	RE
G	SB	LD	IW	PU	CY	P1	P0	DA	FO	HA	CH	RA
H	SB	LD	IW	PU	CY	P1	P0	DA	FO	HA	CH	RA

Supplementary Table S10 List of TaqMan qPCR primer

Gene/Annotation	Primer name	Sequence (5' to 3')	Source
	mouse primer F	5’-CTTGATGTATGAAGGCTTTGG-3’	Sangon Biotech
mouse ACTB	mouse primer R1	5’-TTTGTGTAAGGTAAGGTGTGC-3'	Sangon Biotech
	mouse probe	[5’6-FAM] AGGCCAGCCCTGGCTGCCTC [3'6-TAMRA]	Genscript
	human primer F	5’-CAACTTGAGATGTATGAAGGC-3’	Sangon Biotech
human ACTB	human primer R1	5’-AAGGTGTGCACTTTTATTCAAC-3’	Sangon Biotech
	human probe	[5’6-FAM] AGGTAAGCCCTGGCTGCCTC [3'6-TAMRA]	Genscript
RT-R2	primer R2	5’-CACTCTTTCCCTACACGACG-3’	Sangon Biotech
Ligation-R3	primer R3-test-1	5'-GTACGTTTTAGGCGACTCACT-3'	Sangon Biotech
Ligation-R3	primer R3-test-2	5’-AGTGGTATCAACGCAGAGT**AC**-3’	Sangon Biotech
Ligation-R3	primer R3-test-3	5'-GCAGTGGTATCAACGCAGAG-3'	Sangon Biotech

REFERENCE

1 Han, X. *et al.* Mapping the Mouse Cell Atlas by Microwell-Seq. *Cell* **172**, 1091-1107 e1017, doi:10.1016/j.cell.2018.02.001 (2018).

2 Chen, J. *et al.* PBMC fixation and processing for Chromium single-cell RNA sequencing. *J. Transl. Med.* **16**, 198, doi:10.1186/s12967-018-1578-4 (2018).

3 Hagemann-Jensen, M. *et al.* Single-cell RNA counting at allele and isoform resolution using Smart-seq3. *Nat. Biotechnol.* **38**, 708-714, doi:10.1038/s41587-020-0497-0 (2020).

4 Hughes, T. K. *et al.* Second-Strand Synthesis-Based Massively Parallel scRNA-Seq Reveals Cellular States and Molecular Features of Human Inflammatory Skin Pathologies. *Immunity* **53**, 878-894 e877, doi:10.1016/j.immuni.2020.09.015 (2020).

5 Dobin, A. *et al.* STAR: ultrafast universal RNA-seq aligner. *Bioinformatics* **29**, 15-21, doi:10.1093/bioinformatics/bts635 (2013).

6 Fang, R. *et al.* Comprehensive analysis of single cell ATAC-seq data with SnapATAC. *Nat. Commun.* **12**, 1337, doi:10.1038/s41467-021-21583-9 (2021).

7 Langmead, B. & Salzberg, S. L. Fast gapped-read alignment with Bowtie 2. *Nat. Methods* **9**, 357-359, doi:10.1038/nmeth.1923 (2012).

8 Ramirez, F. *et al.* deepTools2: a next generation web server for deep-sequencing data analysis. *Nucleic Acids Res.* **44**, W160-165, doi:10.1093/nar/gkw257 (2016).

9 Zhang, Y. *et al.* Model-based analysis of ChIP-Seq (MACS). *Genome Biol.* **9**, R137, doi:10.1186/gb-2008-9-9-r137 (2008).

10 Quinlan, A. R. & Hall, I. M. BEDTools: a flexible suite of utilities for comparing genomic features. *Bioinformatics* **26**, 841-842, doi:10.1093/bioinformatics/btq033 (2010).

11 Yu, G., Wang, L. G. & He, Q. Y. ChIPseeker: an R/Bioconductor package for ChIP peak annotation, comparison and visualization. *Bioinformatics* **31**, 2382-2383, doi:10.1093/bioinformatics/btv145 (2015).

12 Stuart, T. *et al.* Comprehensive Integration of Single-Cell Data. *Cell* **177**, 1888-1902 e1821, doi:10.1016/j.cell.2019.05.031 (2019).

13 Jung, M. *et al.* Unified single-cell analysis of testis gene regulation and pathology in five mouse strains. *eLife* **8**, doi:10.7554/eLife.43966 (2019).

14 Wolf, F. A. *et al.* PAGA: graph abstraction reconciles clustering with trajectory inference through a topology preserving map of single cells. *Genome Biol.* **20**, 59, doi:10.1186/s13059-019-1663-x (2019).

15 Zheng, G. X. *et al.* Massively parallel digital transcriptional profiling of single cells. *Nat. Commun.* **8**, 14049, doi:10.1038/ncomms14049 (2017).

16 Cao, J. *et al.* The single-cell transcriptional landscape of mammalian organogenesis. *Nature* **566**, 496-502, doi:10.1038/s41586-019-0969-x (2019).
